# WORKPLACE RETENTION FRAMEWORKS FOR NURSING IN LONG-TERM CARE: AN INTEGRATIVE REVIEW

**DOI:** 10.1093/geroni/igae098.3932

**Published:** 2024-12-31

**Authors:** Winnie Sun, Lucas Martignetti, Jen Calver

**Affiliations:** Ontario Tech University, Lindsay, Ontario, Canada; Ontario Tech University, OSHAWA, Ontario, Canada; Ontario Tech University, Oshawa, Ontario, Canada

## Abstract

**Background:**

Nurses and Personal Support Workers are essential for care and services provided for Long term Care (LTC) homes. Concerns regarding unsafe living and working condition resulting in high turnover rates. Despite high turnover rate, very little guidance to help improve staffing stability and workforce retention planning. The purpose of this integrative review is to synthesise the existing literature regarding LTC staff retention to guide the development of a comprehensive framework addressing LTC staffing issues.

**Methods:**

This review was conducted using Whittemore and Knafl’s integrative review methodology to assess both empirical, theoretical and grey literature. Databases were searched such as CIHNL, PubMed, PyschINFO. Included articles are published in English; use empirical and theoretical methodologies; and focuses on workplace retention frameworks, models, and strategies for the LTC workforce. Articles involving non-facility based LTC and those focused on recruitment rather than retention was excluded. Thematic analysis using Braun & Clark was used to identify themes related to retention frameworks and models influencing turnover.

**Results:**

A total of 1196 articles were retrieved, with 1086 articles screened, while 17 articles were included in the final sample. Examples of identified models including Huber’s Structural Model and the Dual-Driver Model. Retention framework focuses on five group factors: Organizational and personal characteristics, leadership/management, social systems and labour market conditions.

**Conclusion:**

The results of this study will be useful to guide subsequent stages of this research to gain consensus of LTC staff using Delphi methodology to support the co-creation of a workforce retention framework in LTC sector.

